# ‘Supporting the Support Staff’: A Narrative Review of Nutritional Opportunities to Enhance Recovery and Wellbeing in Multi-Disciplinary Soccer Performance Staff

**DOI:** 10.3390/nu16203474

**Published:** 2024-10-14

**Authors:** Christopher Curtis, Christopher Carling, Edward Tooley, Mark Russell

**Affiliations:** 1 School of Pharmacy & Nutrition, University of Navarra, 31009 Pamplona, Spain; ccurtis@unav.es; 2 French Football Federation Research Centre, 78120 Clairefontaine-en-Yvelines, France; ccarling@fff.fr; 3 Sport, Expertise and Performance Laboratory (EA 7370), INSEP, 75012 Paris, France; 4 Drink Sleep Inc. Limited, 8 Walmsley Court, Clayton le Moors, Accrington BB5 5JQ, UK; ed@etnutrition.co.uk; 5 School of Social and Health Sciences, Leeds Trinity University, Leeds LS18 5HD, UK

**Keywords:** recovery, performance, soccer, mental health, health & wellbeing, fatigue, burnout, staff

## Abstract

Background: With ever-increasing training, match-play and travel demands in professional soccer, recovery is vital for athletic performance, a statement amplified in tournament and in-season scenarios. However, alongside supporting the tasks associated with these increased demands, the recovery and wellbeing strategies recommended for playing staff are often unavailable to their support staff counterparts, who routinely experience extended working hours over and above scheduled player attendance. Methods: Focusing on the contributions of nutrition to this undoubtedly multifactorial issue, this narrative review aimed to (1) identify potential strategies to enhance recovery and wellbeing in multi-disciplinary soccer support staff and (2) highlight future research opportunities exploring the benefits of nutrition for those staff in soccer performance-related support roles. Results: The potential health and wellbeing consequences of chronic sub-optimal practices suggest that chrononutrition strategies may be an area of future interest. Notably, nutritional strategies that enhance sleep hygiene and immune function warrant consideration. Individualizing such strategies to maximize recovery and wellbeing in multi-disciplinary soccer support staff should offer an adjunct and complementary strategy to the holistic performance-focused support provided to professional soccer players. Conclusions: Policymakers responsible for organizational and club structures aligned with soccer performance could consider ‘Supporting the Support Staff’ when seeking to improve overall performance.

## 1. Introduction

It is now commonplace for professional soccer clubs to compete in upwards of 50 matches during a ~40-week competitive season, and with successful cup progressions, this total could exceed 70 games [1]. Clubs commonly participate in multiple matches (two to three) within a weekly micro-cycle [2,3,4]. Similarly, within an international tournament-format competition, matches are played approximately every 2–3 days across a ~4–6 week period and are often preceded by prolonged training camps and periods of fixture congestion [5,6]. Moreover, for international squads, major tournaments usually commence after the start of the off-season, thus extending the number of weeks of the year in which players are required to be match-ready (albeit while acknowledging that some degree of delayed start to the subsequent season likely occurs). Collectively, and when combined with pre-season (sometimes incorporating international friendlies), in-season travel demands (sometimes incorporating air travel across time zones and the associated jet lag), varying kick-off times (between 12:00 h and 21:00 h) and minimal recovery periods between fixtures (48–72 h), an increased risk of physical and mental fatigue exists in players, which may also contribute to an increased risk of injury [7,8,9,10,11,12,13,14]. However, while characterization of the influence of soccer training and match-play (including both domestic and international matches) on players is commonplace, the congruent effects on the multi-disciplinary support staff who provide wrap-around health, performance and recovery support to these players are unknown. This is somewhat surprising given the extended work durations that multi-disciplinary support staff experience daily over and above times of player attendance while planning for, undertaking and following up (including data analysis and report writing) player presence at training and competitive grounds. Recently, it has been highlighted by the Fédération Internationale des Associations de Footballeurs Professionnels (FIFPRO) that football players have the equivalent of less than one full day off per week [1]. Given the nature of the multi-disciplinary services provided to these athletes around these demands, it could be hypothesized that coaching and soccer support staff are exposed to, at worst, less time off or, at best, similar working conditions, with less than one day off per week.

Despite scarce research, Calleja-González et al. [15] have proposed that multi-disciplinary sports science support staff may experience common stressors including increased travel demands, fatigue (physical and cognitive), sleep disorders and emotional stress [15]. Notably, among high-performing coaches and multi-disciplinary support staff, the rates of fatigue and mental health symptoms range from 39–55%, including very high psychological distress (14–28%), prevalence of stress-related disorders (~70%) and sleep disturbances (~18%) [16,17,18,19,20,21]. Specific to nutrition, disrupted and rapid eating patterns, as experienced within the associated demands of professional soccer, demonstrate poorer health outcomes in medical professionals [22]. Given the importance of coaching and multi-disciplinary support staff to the tasks associated with the duty of care to professional soccer players, it is noteworthy that these areas of recovery and wellbeing research (including nutritional support) for coaching and performance staff in professional and international soccer seem to have been neglected. Due to the absence of established literature specifically in this area, a systematic literature review was not possible. Therefore, the purpose of this narrative review was as follows: (1) to highlight specific nutrition strategies to potentially optimize wellbeing and recovery in multi-disciplinary soccer support staff and (2) to offer a commentary on potential avenues of future research that seek to optimize nutritional strategies among multi-disciplinary soccer support staff.

## 2. Demands Placed on Multi-Disciplinary Soccer Support Staff in Domestic and Tournament Scenarios

The role-specific demands of multi-disciplinary support staff are not well understood within research [15], with individual roles having nuances between disciplines, clubs and countries. That being said, player schedules within a typical domestic season and tournament scenario, which could indirectly provide some indication as to the minimal corresponding demands placed on multi-disciplinary soccer support staff, have been documented [5,6]. Table 1 provides a typical overview of a training and match schedule for a Premier League soccer team. Within this schedule, allocated days off for specific players are indicated (see Table 1). Given the different training schedules of wider squad players (e.g., injured or loan players), and combined with other role-specific duties (e.g., schedule and program planning, staff meetings, report writing, follow-up consultations, performance analysis, physiotherapy or massage sessions, recovery or rehabilitation sessions, etc.), it is not uncommon for multi-disciplinary soccer support staff to work on allocated player days off, and subsequently to miss out on these opportunities for recovery and wellbeing interventions.

Similarly, in tournament scenarios, training camps and fixtures are congested into a fixed period with limited opportunities for rest for either players or multi-disciplinary soccer support staff [4]. Table 2 provides insight into the demands of international tournaments, where, particularly once the tournament commences, no days off were scheduled. Whilst the overview in Table 2 is specific to the team observed [5], similarly to a domestic soccer season (Table 1), it could be hypothesized that exposures to stressors and the demands placed upon multi-disciplinary soccer support staff during tournament scenarios are heightened due to the ‘enclosed’ environment and nature of tournament formats. This may result in the potential for longer working days, with late-night staff meetings and demands for data and reports from daily activities, potentially leading to increased stressors (e.g., irregular eating patterns, disrupted sleep patterns, etc.) which can be role-specific. For example, it has been proposed by Calleja-González et al. [15] that the demands and stressors of the sport and data scientists in the multi-disciplinary support staff may differ from those of the medical support staff (sport/data scientists: eye health, carpal tunnel syndrome, etc. vs. medical staff: psychological exhaustion) [15]. In pursuit of better understanding of these demands, it may be pertinent for future research to consider monitoring the role-specific requirements of multi-disciplinary soccer support staff during a competitive season and congested fixture periods so that interventions can be more meaningful.

## 3. Responses to Demands Placed on Multi-Disciplinary Soccer Support Staff in Domestic and Tournament Scenarios

### 3.1. Mental Health and Wellbeing

In recent years there has been increased research and awareness of mental health and wellbeing in sport; however, the main focus of this research has been on athletes themselves [16,23]. Accordingly, limited research is available concerning the mental health and wellbeing of coaches and support staff [16,24,25]. Notably, Leprince et al. [16] and Norris et al. [26] identify a range of stressors that have been classified as specific to coaches such as (but not limited to) organizational factors, player injury and performance, contextual experiences and inter/intra-personal experiences (e.g., team dynamics, communication and self-reflection), and which may influence mental health and wellbeing. Whether these stressors are the same for additional multi-disciplinary soccer staff outside of coaches (i.e., medical, physiotherapy, strength and conditioning, nutrition, etc.) remains to be investigated. Of the limited research available, approximately one third of coaches and multi-disciplinary support staff (both ~35%) have reported that they had sought treatment for a psychological issue or mental health problem [17]. Similarly, when compared against an athlete sample, multi-disciplinary support staff scored worse for both the anxiety/insomnia subscale and social dysfunction [17]. Specifically for soccer, investigations of strategies that optimize coach mental health and wellbeing are starting to be investigated. For example, the French Football Federation (FFF) Research Centre has begun to examine the mechanisms involved in the deterioration of the mental health and wellbeing of coaches within their organization, looking at longitudinal monitoring (e.g., across a season) and multifactorial factors that influence coach wellbeing (e.g., environmental, personal and relational). For more details of the research being conducted by the FFF into coach mental health and wellbeing, readers are directed to Leprince et al. [16]. Nevertheless, it could be suggested that the recovery requirements and subsequent strategies may differ between multi-disciplinary soccer support staff, depending on their role within the club. Therefore, with a view to improving personalized and targeted nutritional recovery strategies between intensive soccer-related periods (e.g., congested fixtures or tournament formats) and role-specific functions, identifying specific stressors of mental health and wellbeing among multi-disciplinary soccer support staff may be an avenue for future investigation.

### 3.2. Sleep Hygiene

Moderate to severe sleep disturbances have previously been reported among coaching and multi-disciplinary support staff [17,27], in spite of such staff members having adequate sleep hygiene knowledge [28]. It has been previously documented that specific areas of sleep hygiene may warrant further education [15], as this has been shown to increase positive changes in sleep patterns and behaviors [29,30]. Specific to soccer, less total sleep time, time spent in bed and worse quality sleep following late-night soccer matches and international competition have been documented in soccer players [31,32]. However, corresponding research profiling the effects on multi-disciplinary soccer support staff as opposed to players remains unclear. Based upon the previous sleep disruptions observed in coaching and multi-disciplinary support staff [17], when combined with the similar experiences of soccer players highlighted in Section 2 of this article (e.g., lack of days off, travel demands, late-night matches, schedules disruption, etc.), we surmise that comparable sleep responses may be observed in multi-disciplinary staff. Studies involving the addition of quantitative measures of sleep within these cohorts are required to confirm such a statement.

Sleep hygiene practices including (but not limited to) the promotion of light–dark cycles, filtering short wavelengths prior to sleep and some nutritional strategies have been documented in soccer players [33,34]. However, despite the potentially positive findings of increasing sleep education and sleep hygiene improvement strategies, in a practical sense, the implementation of these may be compromised in multi-disciplinary soccer support staff due to demanding or ‘out of hours’ schedules (e.g., late-night team meetings, irregular eating times), the negative influences of travel, potential time zone changes and unfamiliar surroundings (e.g., team hotel, training camp, etc.). Due to these factors, it could be hypothesized that multi-disciplinary soccer support staff are also at risk of suppression to their immune system [15], although further research is necessary to confirm this. There is a link between the recuperative effects of sleep on immune function [34]; therefore, these factors need to be considered within recovery strategy development, as well as how these factors, alongside immune system suppression, influence both psychological and physiological markers among multi-disciplinary soccer support staff.

## 4. Future Directions of Nutrition Research to Enhance Recovery and Wellbeing in Multi-Disciplinary Soccer Support Staff

Given the role-specific stressors and responses to these stressors (identified in Section 2 and Section 3) that coaches and multi-disciplinary soccer support staff are likely to encounter [15,16,26], targeting nutritional strategies that focus on the maintenance and support of immune health and improving sleep hygiene seem logical when seeking to optimize recovery and wellbeing and should be undertaken on a case-by-case basis (taking into account factors such as environment and logistics) across domestic (i.e., pre- and in-season training and competition periods) and international tournament scenarios.

### 4.1. Nutrition and Immune Health

As identified, coaches and multi-disciplinary soccer support staff will likely be exposed to a number of environmental and psychological stressors, which are known contributors to compromised immune function [35]. Despite being undertaken in athletic populations, research has identified multiple immune health risk factors, including (but not limited to) intensified training in the winter; long-haul travel; low energy availability; and high levels of psychological stress and anxiety [36]—factors to which multi-disciplinary soccer support staff may also have high exposure, given the demands of professional soccer [1,7] and the increasing demands involved in supporting players. Impaired immune (salivary immunoglobulin A; s-IgA) and stress (salivary cortisol; sCort) responses have been documented in professional soccer players following a 7-day training period [37] and across nine competitive fixtures [38]. Likewise, decreased s-IgA following high-intensity training and match-play has been observed, while increased sCort occurred in starting players from pre- to post-match. However, acknowledging the absence of playing and training demands for support staff, such physiological responses indicate a potential role for such markers when profiling the immune and stress responses to various stressors inducing physiological perturbations. Accordingly, future research opportunities exist to complement previous qualitative work with physiological insight regarding the health and wellbeing of support staff. Incorporating the use of technology (e.g., heart rate variability and activity monitoring devices, etc.), alongside immune-associated biomarkers (e.g., s-IgA, sCort, etc.) may provide some additional insight into the physiological responses within these cohorts and allow for further targeted nutrition interventions to help support soccer support staff during both in-season and tournament formats.

Albeit for athletic populations, Walsh [39] has proposed a model of ‘resistance’ and ‘tolerance’ within immune function. Interested readers are directed to this review [39] for further information. From a nutritional perspective, within the Walsh review [39], a range of nutritional strategies have been proposed for immune tolerance, including zinc, glutamine, carbohydrates, bovine colostrum, beta-glucans, echinacea and caffeine, and immune resistance, including probiotics, vitamins C, D and E, polyphenols and omega-3 [39]. Examples of foods offering these nutrients can be seen in Table 3. Given the large volume of existing research into nutrition and immune function among both general and athletic populations, the proposed strategies offered by Walsh [39] present unique opportunities to investigate nutrition strategies and the optimization of recovery among multi-disciplinary soccer support staff, especially when multiple as opposed to singular strategies are used in combination.

Furthermore, an area of developing interest, albeit within general populations, in optimizing immune function is the interaction between gut microbiota and the immune system [40,41]. It has been proposed that metabolites from gut microbiota such as short-chain fatty acids (SCFAs), tryptophan metabolites and bile acid metabolites promote the differentiation and function of immune-suppressive cells and inhibit inflammatory cells [40]. To the best of the authors’ knowledge, this research is lacking in sport-specific contexts. However, given their proposed mechanisms and benefits within general populations, dietary strategies such as promoting the consumption of dietary fiber to promote fermentation in the intestines (and subsequent production of SCFAs) and dietary protein intakes (to increase intakes of amino acid sources involved in digestive and biochemical processes associated with tryptophan and bile acid metabolism) may warrant future interest among multi-disciplinary soccer support staff, particularly as such strategies assist in the interaction between gut microbiota and the immune system [40]. From an applied perspective, multi-disciplinary soccer support staff may wish to consider consuming food sources rich in vitamins and minerals, polyphenolic compounds, pre- and probiotics and omega-3 (sources of which can be found in Table 3) to help with both immune tolerance and immune resistance, as proposed by Walsh [39].

### 4.2. Nutrition and Sleep Hygiene

Despite research indicating some proposed nutrition strategies to improve sleep within athletic cohorts [33,34], there is little documented research to indicate how such strategies can help with the recovery and wellbeing of multi-disciplinary support staff in applied environments. Given the identified role-specific stressors relating to domestic and international soccer demands (identified within Section 2) and the proposed responses (highlighted within Section 3), improving sleep hygiene practices via the use of sleep-monitoring devices that track metrics such as total sleep time, sleep phase, etc. and targeted nutrition strategies may provide some benefits to recovery and wellbeing for multi-disciplinary soccer support staff during domestic and international tournament formats.

It has been identified that several neurotransmitters modulate the sleep–wake cycle. These neurotransmitters include (but are not limited to) 5–hydroxytryptophan, gamma-aminobutyric acid, orexin, melanin concentrating hormone, cholinergic, galanin, noradrenaline and histamine [42,43]. Ostensibly, any nutrition strategy that influences these neurotransmitters may positively influence sleep [42,43]. A review into sleep, nutrition interactions and their implications for athletes was conducted by Doherty et al. [42], which identified various potential nutrition strategies that may enhance sleep including carbohydrate intakes (including the timing and type), melatonin, tryptophan-rich protein, antioxidants (namely vitamins A, C and E), tart cherry, B-vitamins and magnesium (Table 3). With existing research into nutrition and sleep hygiene, combined with the proposed dietary strategies to enhance sleep in athletic populations suggested by Doherty et al. [42], a unique opportunity exists to investigate sleep-enhancing-specific nutrition strategies and the optimization of recovery and wellbeing for multi-disciplinary soccer support staff around in-season and international tournaments, particularly in view of the reported sleep disturbances amongst coaching and multi-disciplinary support staff [17,27]. Therefore, multi-disciplinary support staff may wish to consider foods rich in these compounds to potentially aid in sleep enhancement. Interestingly, Doherty et al. [42] also suggest alcohol consumption may negatively influence sleep, due to potential changes in circadian rhythms. It could be hypothesized that due to demands on multi-disciplinary soccer support staff (highlighted in Section 2 of this review), alcohol consumption and sleep disruption may be a factor in sleep hygiene, although further investigation within these cohorts to substantiate this is required.

**Table 3:** Examples of nutrients to assist with immune tolerance, immune resistance and sleep. Adapted from Walsh [39] and Doherty et al. [42].

Immune Tolerance: Walsh [39]	
Nutrient	Examples of Food Sources (non-exhaustive)
Zinc	Oysters, Red Meat, Spinach, Zinc Lozenges
Glutamine	Wholegrains, Poultry, Fish
Bovine Colostrum	Cows’ Milk
Beta-glucans	Cereals—Oats, Barley, Fungi (Mushrooms), Seaweed
Echinacea	Echinacea Herb Supplementation
Caffeine	Tea, Coffee, Green Tea, Dark Chocolate
**Immune Resistance: Walsh** **[39]**	
Probiotics	Yogurt, Kefir, Kombucha, Sauerkraut
Vitamin D	Oily Fish, Eggs, Fortified Cereals
Omega-3	Oily Fish, Soybean, Chia Seeds
**Sleep Hygiene: Doherty et al.** **[42]**	
Vitamin A	Eggs, Oily Fish, Carrots, Leafy Green Vegetables
Vitamin B complex	Meat, Fish, Eggs, Oats, Milk, Fortified Cereals
Magnesium	Leafy Green Vegetables, Legumes, Nuts and Seeds
Melatonin	Cows’ Milk
Tryptophan-rich Protein	Milk, Poultry, Fish, Eggs, Cheese, Leafy Green Vegetables
**Immune & Sleep Functions; Walsh** **[39]** **& Doherty et al.** **[42]**	
Carbohydrate	Bread, Cereals, Grains, Pasta, Potatoes, Rice
Vitamin C	Citrus Fruits, Broccoli, Kale, Peppers
Vitamin E	Almonds, Sunflower Seeds, Avocados, Kiwi Fruit
Polyphenols	Berries, Cocoa, Vegetables, Flaxseed, Tart Cherry

### 4.3. Chrononutrition Research—An Opportunity to Combine Health, Sleep and Nutrition Monitoring of Multi-Disciplinary Soccer Support Staff?

Given the demands and responses to both domestic and tournament scenarios for multi-disciplinary soccer support staff, an area of future research direction when seeking to optimize recovery strategies within these cohorts may be the field of chrononutrition. Chrononutrition is the study of relationships between the circadian process and food intake [44] and has been investigated in a number of health settings [44,45,46]. Some of the key characteristics of poor chrononutrition practices include (but are not limited to) disrupted sleep, poor nutrition timings and/or nutrient composition [46]—factors that are likely commonplace within professional soccer due to fixture scheduling and the associated travel demands (e.g., pre-season tours, European and international fixtures, etc.). These factors, when coupled with the increased and ‘out of hours’ travel demands associated with such fixtures (i.e., international and domestic travel), may have consequences for the implementation of recovery modalities [31,32,47]. Similarly, these impacts are further unknown when factoring in travel involved for multi-disciplinary soccer support staff who, similar to international soccer players, may have ‘dual roles’ at both a domestic club and international teams. Circadian rhythm processes are thought to represent all physiological processes involved in a 24 h cycle, such as (but not limited to) the sleep/wake cycle, blood pressure, heart rate, hormone secretion, cognitive performance and mood regulation [44], factors that may also be beneficial to overall health, but which may have metabolic health implications if continually disrupted [46]. Such misalignment of circadian rhythmicity has been reported to influence food intake, glucose metabolism and weight regulation [45,46,48,49]. Later meal timings and irregular eating (e.g., post-match nutrition after a late kick-off), which are not in line with the biological clock, may be associated with poorer diet-related health outcomes [44,45,46].

Additionally, night work (such as that associated with late kick-off times in professional soccer) is considered one of the negative components strongly correlated with circadian disruption that induces adverse health effects [45,46]. It has previously been demonstrated that the adverse effects of jetlag, which exposes subjects to a light–dark cycle lengthened to 28 h, is out of synchronicity with the endogenous clock, during which time melatonin and body temperature rhythms within a ~24 h period are disrupted. These exposures have shown increased postprandial glucose, insulin and mean arterial pressure, alongside induced decreases in leptin and sleep efficiency, and inversion of the cortisol profile across the behavioral cycle [50]. Given the increasing demands within professional soccer [1,7], these could be considered areas of priority for health and wellbeing with both players and multi-disciplinary soccer support staff.

Several dietary strategies have been proposed to optimize chrononutrition practices, which include (but are not limited to) eating the majority of daily calories and carbohydrates at lunch time and in the early afternoon, avoiding late evening dinners and the timing of food intake during light time vs. evening vs. night [45,46]. The mechanisms for these proposed dietary interventions are based on three different dimensions of eating behavior, namely, timing, frequency and regularity, for the maintenance of metabolic health in relation to insulin resistance and glucose tolerance [45,46]. However, whilst chrononutrition is an advancing science, there remains much to be learnt about the nature and timing of food provision in regulating diets for the purpose of metabolic health [46], a statement that may be of greater pertinence for multi-disciplinary soccer support staff. Such dietary strategies and practices may not be achievable for multi-disciplinary soccer support staff, given the differing training and match-play schedules and the logistics involved within these for travel, disrupted sleep times, etc. Given the increasing congested fixtures, recovery demands and travel within professional soccer, as highlighted by FIFPRO) [1,7], it is perhaps pertinent that the Union of European Football Associations (UEFA) expert group statement on nutrition in elite soccer highlighted the need for further research in this area to optimize nutrition practices across travel, differing time zones and congested fixtures for playing personnel [1,6,7,51], a statement that also could be a consideration in the support of optimizing recovery practices among multi-disciplinary soccer support staff as well.

## 5. Proposed Theoretical Framework for ‘Supporting the Support Staff’: Nutritional Opportunities to Enhance Recovery and Wellbeing in Multi-Disciplinary Soccer Performance Staff

**Figure 1:**
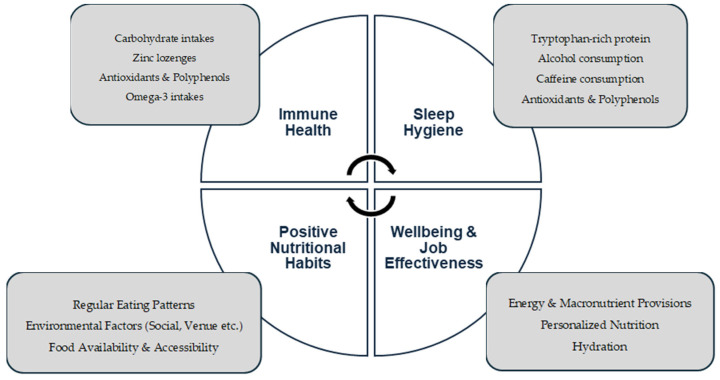
A theoretical framework of nutritional opportunities to enhance recovery and wellbeing in multi-disciplinary soccer performance staff. The framework focuses on key considerations (white circle) and proposed key considerations for practitioners (grey boxes). Directional arrows represent the interlinking between the framework categories.

As already highlighted within this review, scarce research currently exists surrounding the role-specific demands of multi-disciplinary support staff [15]. Nutritional support may be an avenue to assist in supporting staff in recovery and wellbeing strategies. With this in mind, we propose a theoretical nutritional support framework (Figure 1) for sports nutrition practitioners, training ground and stadium staff and (if relevant) personal chefs and catering staff to consider and collaborate on. The premise of this theoretical framework centers on nutritional strategies that have been identified within this review and draw upon strategies related to immune health and sleep hygiene. These areas may also be important factors for consideration when considering the multiple logistical challenges in both in-season and tournament-style scenarios in order for practitioners to aid in ‘Supporting the Support Staff’.

## 6. Conclusions

As highlighted, multi-disciplinary soccer support staff are regularly exposed to risk factors associated with poor health and wellbeing practices within the parameters of congested fixtures (e.g., role-specific stressors, disrupted nutrition timing, poor sleep practices, late travel times, etc.). The effects of these factors on their health and wellbeing are currently not well-investigated. Optimizing these practices and strategies for multi-disciplinary soccer support staff would help aid their recovery and may have a positive outcome for the support provided to players. From an applied perspective, with the increasing use of performance chefs within professional soccer, a nutritional consideration may be for performance nutrition practitioners and club chefs to further collaborate and design menus and provide environments to support a holistic approach (e.g., wellbeing, performance, positive eating habits, etc.) for coaching and support staff (Figure 1). To the best of the authors’ knowledge, currently, there is no chrononutrition-related research within soccer or relating to the sport’s multi-disciplinary support staff. Available studies have looked at recovery modalities in isolation of soccer players, relating to sleep [31,32], nutrition in congested fixtures [6] and whether circadian rhythm influences training performance [47]. Given the multifactorial approaches to identifying chrononutrition practice, this proposal offers a unique opportunity to undertake a multifactorial approach (i.e., investigating physiological, biological, nutritional and subjective markers) of chrononutrition for multi-disciplinary soccer support staff, with a view to further optimizing recovery and health practices, enabling opportunities in ‘Supporting the Support Staff’.

## Figures and Tables

**Table 1:** A typical monthly schedule for a top professional soccer club in the Premier League (adapted from Ranchordas et al. [6]). (Proposed days off indicated in bold).

Month						
Monday	Tuesday	Wednesday	Thursday	Friday	Saturday	Sunday
			Soccer Training	MD-1	Match (League)	Recovery
			Gym			
Match (FA Cup)	Recovery	**DAY OFF**	Soccer Training	MD-1	Match (League)	Recovery
			Gym			
Soccer Training	MD-1	Match (Europe)	Recovery	MD-1	Match (League)	Recovery
Gym						
**DAY OFF**	MD-1	Match (EFL Cup)	Recovery	MD-1	Match (League)	Recovery
**DAY OFF**	MD-1	Match (Europe)	Recovery	MD-1	Match (League)	

EFL = English Football League; FA = Football Association; MD-1 = Match-Day minus one (Light Training).

**Table 2:** Team schedule during the training period and the tournament (adapted from Wilke et al. [5]. (Proposed days off indicated in bold).

Week 1 (Training)						
Player Testing	Training	Training	Blood Samples	Match (Friendly)	**DAYS OFF (×3)**	
			Training			
**Week 2 (Training)**						
Training Camp (specific details not provided within Wilke et al. [4]					Travel to Tournament	
**Week 3 (Tournament)**						
Training	Training	Training	Training	Training	Match	Recovery
**Week 4 (Tournament)**						
Match	Recovery	Match	Recovery	Match	Recovery	Training
**Week 5 (Tournament)**						
Training	Match	Recovery	Training	Match	Recovery	Training
**Week 6 (Tournament)**						
Match	Recovery	Training	Match	Recovery	Training	Match
